# A Hidden Markov Model for Single Particle Tracks Quantifies Dynamic Interactions between LFA-1 and the Actin Cytoskeleton

**DOI:** 10.1371/journal.pcbi.1000556

**Published:** 2009-11-06

**Authors:** Raibatak Das, Christopher W. Cairo, Daniel Coombs

**Affiliations:** 1 Department of Mathematics and Institute of Applied Mathematics, University of British Columbia, Vancouver, British Columbia, Canada; 2 Alberta Ingenuity Centre for Carbohydrate Science, Department of Chemistry, University of Alberta, Edmonton, Alberta, Canada; UT Southwestern Medical Center, United States of America

## Abstract

The extraction of hidden information from complex trajectories is a continuing problem in single-particle and single-molecule experiments. Particle trajectories are the result of multiple phenomena, and new methods for revealing changes in molecular processes are needed. We have developed a practical technique that is capable of identifying multiple states of diffusion within experimental trajectories. We model single particle tracks for a membrane-associated protein interacting with a homogeneously distributed binding partner and show that, with certain simplifying assumptions, particle trajectories can be regarded as the outcome of a two-state hidden Markov model. Using simulated trajectories, we demonstrate that this model can be used to identify the key biophysical parameters for such a system, namely the diffusion coefficients of the underlying states, and the rates of transition between them. We use a stochastic optimization scheme to compute maximum likelihood estimates of these parameters. We have applied this analysis to single-particle trajectories of the integrin receptor lymphocyte function-associated antigen-1 (LFA-1) on live T cells. Our analysis reveals that the diffusion of LFA-1 is indeed approximately two-state, and is characterized by large changes in cytoskeletal interactions upon cellular activation.

## Author Summary

Many important biological processes begin when a target molecule binds to a cell surface receptor protein. This event leads to a series of biochemical reactions involving the receptor and signalling molecules, and ultimately a cellular response. Surface receptors are mobile on the cell surface and their mobility is influenced by their interaction with intracellular proteins. We wish to understand the details of these interactions and how they are affected by cellular activation. An experimental technique called single particle tracking (SPT) uses optical microscopy to study the motion of cell-surface receptors, revealing important details about the organization of the cell membrane. In this paper, we propose a new method of analyzing SPT data to identify reduced receptor mobility as a result of transient binding to intracellular proteins. Using our analysis we are able to reliably differentiate receptor motion when a receptor is freely diffusing on the membrane versus when it is interacting with an intracellular protein. By observing the frequency of transitions between free and bound states, we are able to estimate reaction rates for the interaction. We apply our method to the receptor LFA-1 in T cells and draw conclusions about its interactions with the T cell cytoskeleton.

## Introduction

The lateral mobility of cell-surface proteins plays a critical role in mediating the biological functions of membrane proteins [1]. The diffusion of membrane components is affected by factors including the viscosity of the membrane, clustering of the receptor, and binding to cellular components. The spatio-temporal dynamics of membrane-associated receptors are therefore of considerable interest as they can provide crucial insight into cellular signal transduction. A variety of biophysical techniques, particularly fluorescence microscopy experiments, have been extensively utilized to quantify the lateral mobility of membrane proteins. The complementary techniques of single particle tracking (SPT, reviewed in Ref. [2]) and fluorescence recovery after photobleaching (FRAP, reviewed in Ref. [3],[4]) probe these dynamics at different length scales. FRAP captures the behavior of a population of labeled particles on a spatial scale of a few microns, while SPT records the dynamics of individual molecules or small macromolecular clusters over lengths of tens to hundreds of nanometers. In a typical SPT experiment, a membrane-associated protein is labeled, either fluorescently or with an antibody conjugated bead, and imaged using high speed video microscopy with a temporal resolution of tens of milliseconds or less. The spatial coordinates of the particle can be determined to a sub-optical resolution of tens of nanometers, permitting a detailed examination of the particle's motion [5],[6]. The enhanced spatial resolution of SPT, as well as its non-ensemble nature, make the technique attractive for detailed single molecule studies of cell surface receptor dynamics.

The analysis of particle trajectories is commonly based on a classification into different modes of motion, such as Brownian, hop diffusion, confined motion or directed diffusion based on fits to their mean squared displacement (MSD) over time [7],[8]. Brownian diffusion is characterized by a linear increase in MSD with time with a slope proportional to the diffusion coefficient. The timescale of diffusion is often treated by analyzing diffusion over short time periods (typically 1–4 timesteps or tens of milliseconds), referred to as microdiffusion, or longer time periods (typically on the order of seconds), referred to as macroscopic diffusion. Deviations from linearity are ubiquitous in time versus MSD data for membrane-associated proteins. Such deviations are variously attributed to flow, the presence of obstacles, membrane compartmentalization or changes in membrane lipid organization [9],[10]. Numerous modelling studies have examined the effect of membrane structure on particle trajectories and have proposed methods to identify structural features of the plasma membrane responsible for the observed diffusion [11]–[16]. Further difficulties in the analysis of SPT data arise as individual trajectories often show evidence of heterogeneity that is not easily resolved [17]–[21]. Thus new methods of analyzing particle trajectories are needed to extract and interpret subtle changes in diffusive behavior.

Both FRAP and SPT experiments on adhesion receptors commonly show a large reduction in receptor mobility upon binding with cytoskeletal components. Therefore, receptor motion may involve multiple states (i.e. bound or unbound) that contribute to the diffusion of the receptor in different ways. In a previous study of the T cell integrin receptor, LFA-1, particle trajectories were acquired with a temporal resolution of 1000 frames/s using antibody-conjugated beads [22]. Macroscopic diffusion coefficients calculated using an MSD analysis were shown to be distributed in two distinct subpopulations. Relative contributions of the two subpopulations varied when the cells were treated with different pharmacological agents, and when different conformations of the protein were preferentially labeled. These results suggested a dynamic equilibrium of LFA-1 between two states with distinct mobilities. Using cytoskeletal inhibitors, it was shown that the cytoskeleton was largely responsible for the state with low mobility. The existence of multiple states with distinct diffusive properties has also been observed for the CD2 receptor on the surface of T cells [23]. In these studies, evidence of heterogenous diffusion was obtained using an MSD analysis that required a large number of replicates for a reliable identification of the underlying states. Additionally, the analysis used relied on changes in the average diffusion, making it difficult to detect subtle or transient changes in diffusivity within single trajectories.

Here, we present a novel analytical framework to identify multiple diffusion states and estimate probabilities of switching between them, from particle trajectories of cell-surface proteins. Transitions between these states represent the binding and unbinding of receptors to cytoskeletal contacts or other intracellular signalling components. We introduce a new model that treats particle trajectories as the outcome of a two-state hidden Markov process, parametrized by diffusion coefficients of the two states and rates of transition between them. We adopt a likelihood maximization strategy to identify model parameters that best describe a set of tracks, thus characterizing the underlying diffusive states and the kinetics of the transitions between them.

This analysis was first tested with a series of simulated trajectories and compared with previous approaches for isolating subpopulations. We show that our analysis achieves a more accurate and informative resolution of the underlying biophysical parameters for a complex trajectory consisting of multiple states of diffusion. We tested the applicability of this analysis to experimental data of LFA-1 particle trajectories, and found that the diffusion of this adhesion molecule can indeed be treated as a two-state process due to its interactions with cytoskeletal binding partners. Our analysis identifies the characteristic diffusion coefficient of LFA-1 in the two states, and reveals the kinetics of switching between them. The use of a likelihood-based approach further allowed us to compare multiple models for given experimental data, and identify the statistically most optimal model that captures the receptor dynamics.

## Results

### A two-state hidden Markov model for single particle tracks

We modeled single particle tracks for a labeled, membrane-associated protein that binds to a uniformly distributed intracellular substrate, such as cytoskeletal binding proteins. This binding is schematically represented by the bimolecular reaction
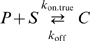
(1)where 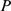 and 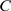 are the free and bound forms of the protein, and 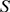 is the substrate. The kinetics of this interaction are characterized by the bimolecular forward rate constant, 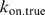, and a first order unbinding rate constant, 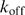. We assume a homogeneous spatial distribution of the substrate so that at equilibrium the binding reaction is effectively first order with a rate constant 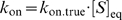, where 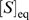 is the equilibrium concentration of the free substrate. With this assumption, we can represent the bimolecular reaction, at equilibrium, by the unimolecular reaction
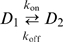
(2)where 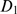 and 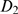 are the diffusion coefficients of the protein in its free and bound forms, respectively. We further make the simplifying assumption that the particle is imaged instantaneously, and that changes in the particle state occur only at the acquisition time, implying that the particle is entirely in one or the other state between successive image frames (see *Discussion* for more details). For a constant frame rate, 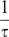, where 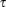 is the sampling interval, this assumption leads to the following fixed transition probabilities for the particle to switch its state between successive frames (see *Text S1* for a derivation):
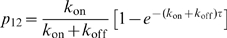
(3)

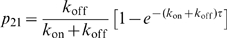
(4)

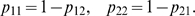
(5)


**Figure 1:**
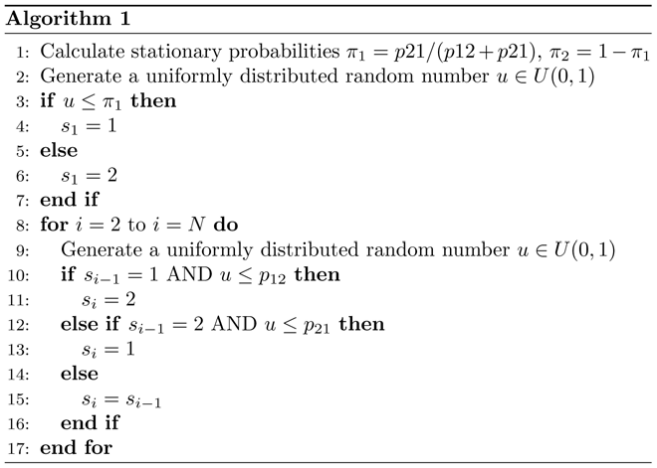
Simulation algorithm for a 2-state Markov chain.

In this model, the state sequence of the particle during an SPT experiment is regarded as a 2-state Markov chain. The displacement of the particle at each step is the outcome of Brownian diffusion with a diffusion coefficient corresponding to the particle state at that interval. As described in *Materials and Methods*, to simulate a single particle track arising from the 2-state dynamics described above, we first generated a discrete Markov chain that specifies the particle state at each time point. The initial state of the particle was chosen randomly according to the stationary probabilities of the two states, and the remaining states were determined using a discrete-time stochastic algorithm (Algorithm 1; Fig. 1). The particle displacements at each frame were sampled from a zero mean Gaussian distribution with variance proportional to the diffusion coefficient.

**Figure 2:**
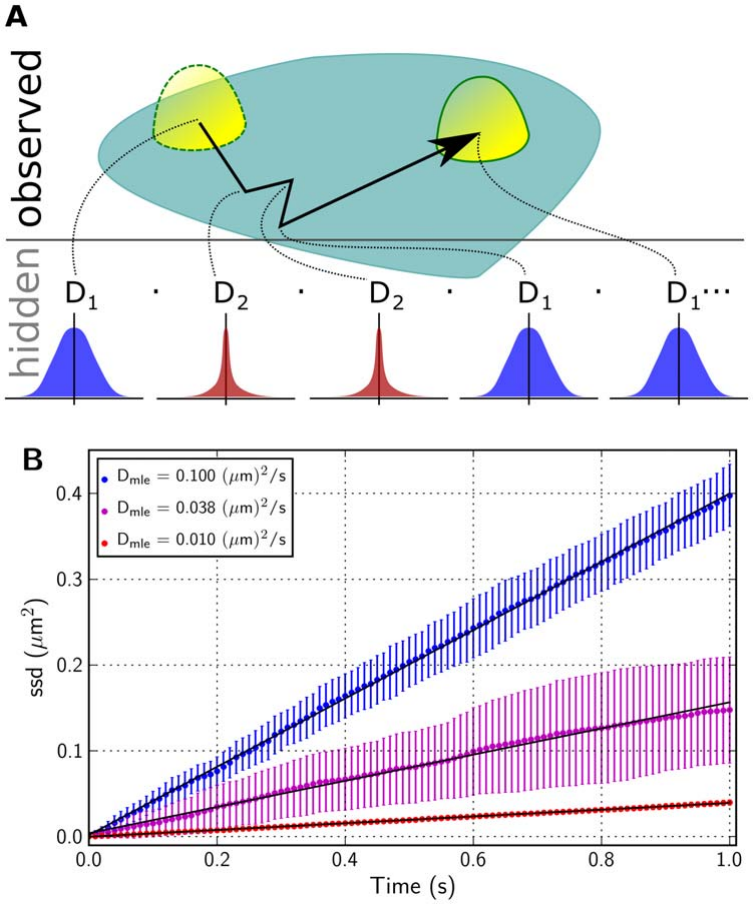
Two-state particle trajectories. (A.) A schematic 2-state particle trajectory consisting of a sequence of observable displacements arising from an underlying state sequence hidden from the observer (B.) Sum of squared displacements (ssd) as a function of time for simulated particle tracks exhibiting purely Brownian motion with a diffusion coefficient 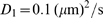, or 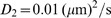, or 2-state motion switching between these two diffusion coefficients with transition probabilities 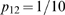 and 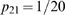. Each ssd trace is generated from a total of 20 independently simulated tracks, each containing 100 frames sampled at 10 ms intervals. The colored symbols mark the mean±standard deviation of the ssd for each set of tracks, and the solid lines are the best linear fits to the time versus mean ssd data.

In an experimental trajectory, only the particle position is recorded and information about the particle state must be inferred from the displacement of the particle between successive frames. Therefore, in our model, a particle trajectory is regarded as the outcome of a 2-state hidden Markov model (HMM) [24] consisting of a sequence of discrete states – free or bound – that are hidden from the observer, and an observable displacement at each time point from a well-defined probability distribution (Fig. 2A). As demonstrated below, a traditional analysis using the mean squared displacements does not reveal the diffusion coefficients of the constituent states, the rates of transition between them, or the state-sequence underlying an observed track. Therefore, we developed a likelihood-based analysis of single particle tracks to infer these model parameters and thus quantify the underlying biophysical process. It should be noted that, though we have chosen to test the two-state model described above, the hidden Markov formulation and the associated likelihood maximization scheme is a more general and powerful technique for analyzing a wide range of models. In particular, for sufficiently well resolved data, an arbitrarily complex model with multiple states, with diffusive, confined or directed motion could be analyzed using this method. We intend to explore such general models in future studies.

### Maximum likelihood parameter estimation

We first consider a trajectory arising from 2D Brownian diffusion and sampled at fixed time intervals, 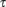. For an observed sequence of independent displacements 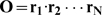, the likelihood of a diffusion coefficient 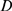 is
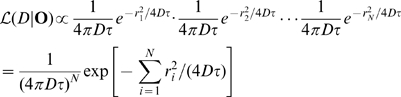
(6)where 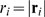. To calculate the maximum likelihood estimate of 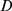 we define the log likelihood function
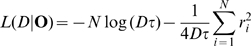
(7)(up to an additive constant) and maximize it with respect to 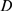 to obtain
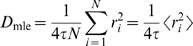
(8)where 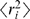 is the mean squared step size. This maximum likelihood estimate of the diffusion coefficient is most closely related to the microscopic diffusion coefficient obtained from an MSD analysis.

The previous equation can be rewritten in the following familiar form
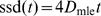
(9)with 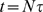 and 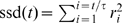 is the sum of squared displacements. For a particle undergoing Brownian diffusion, the single parameter 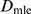 sufficiently describes the particle motion. In Fig. 2B we plot 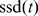 for three sets of simulated trajectories, two for Brownian diffusion with a single diffusion coefficient, and one for 2-state diffusion. We note an excellent linear fit to 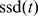 in each case, and an excellent match between the estimated 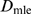 and 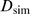 for the Browmian diffusion trajectories.

For the 2-state system described above, as the track length increases, 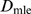 approaches an effective diffusion coefficient, 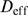, defined as the weighted average of the diffusion coefficient in each state. For a sufficiently long track, or when averaging over multiple tracks, the particle is in state 1 for a fraction of steps 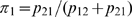, and in state 2 for a fraction of steps 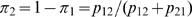. Thus, the expected value of 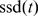 is
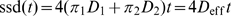
(10)


The slope of a linear fit to 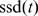 for the 2-state tracks in Figure 2B is indeed this weighted average for the chosen set of parameter values. This 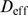 is a good descriptor for the overall mobility of a 2-state particle, but it does not reveal the underlying diffusion coefficients and their relative contributions. We now describe a likelihood maximization scheme to identify these parameters by fitting particle tracks to a 2-state hidden Markov model.

The 2-state HMM is characterized by two diffusion coefficients and two transition probabilities. We parametrized the model by the parameter set 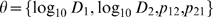, and sought to calculate the likelihood of 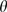, for an observed particle track 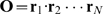


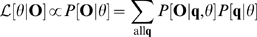
(11)where 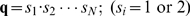 represents a particular state sequence of the Markov chain. The probability 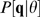 of observing the state sequence depends only on the two transition probabilities, whereas, for that state sequence, the probability 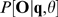 of observing the track depends only on the two diffusion coefficients. Because the possible number of state sequences grows exponentially with the number of steps in a track, a direct calculation using the above equation is computationally prohibitive. However, the forward-backward algorithm [25],[26] efficiently calculates this probability by recursively evaluating the forward variable 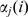, defined as the probability of observing the partial sequence of steps 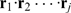 up to step 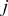, and being in state 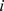 at step 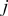, given the model parameters 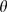:
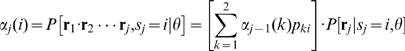
(12)


The probability of observing a track for a given choice of the parameters 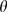 is
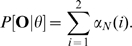
(13)


**Figure 3:**
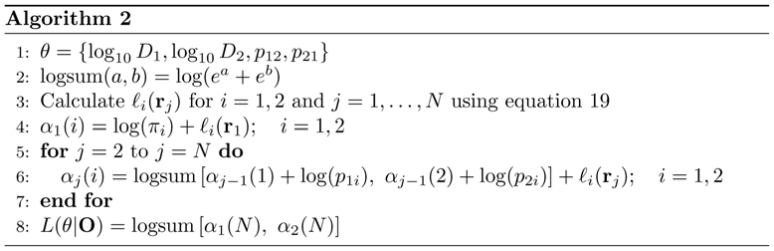
Forward algorithm for calculating log likelihood of parameter values of a 2-state HMM for a given track 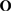.

As described in *Materials and Methods*, we used a modified version of the forward algorithm to calculate the log likelihood of the parameter set 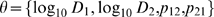 for an observed set of particle tracks (Algorithm 2; Fig. 3).

**Figure 4:**
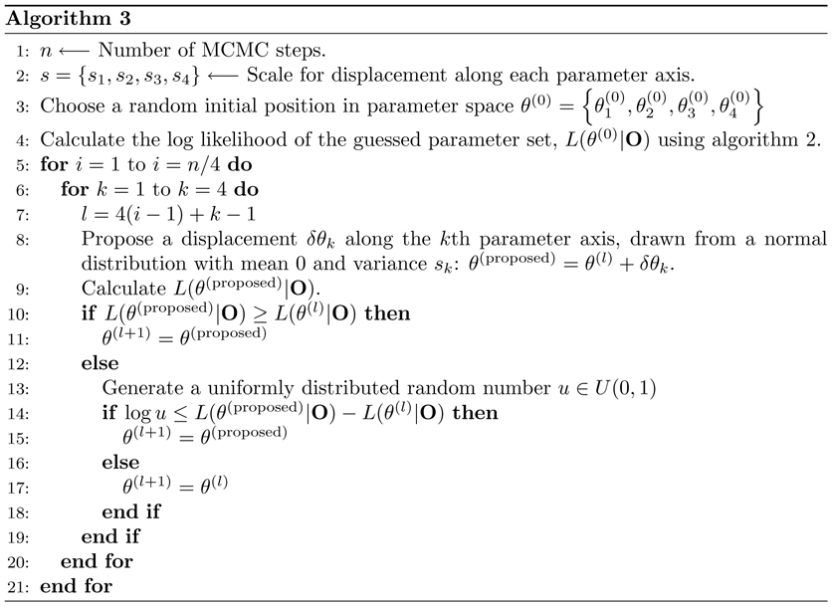
Algorithm for MCMC maximization of the log likelihood function 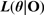 with respect to the model parameters 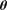.

**Figure 5:**
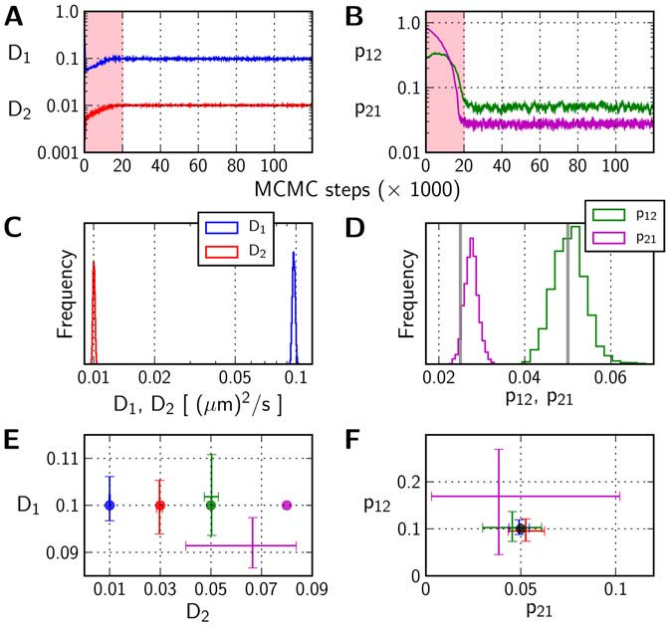
Parameter optimization for two-state model. A typical MCMC parameter optimization for an ensemble of 20 simulated 2-state particle tracks with model parameters 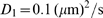, 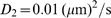, 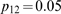 and 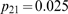. Each track consists of 1000 frames sampled at 1 ms intervals. (A., B.) HMM parameter values are plotted for an MCMC trajectory that starts with a random initial guess and stochastically evolves in the parameter space according to Algorithm 3 (Fig. 4). The shaded part of the plots indicate the burn-in phase during which the trajectory approaches the log likelihood maxima. (C., D.) Histogram of parameter values from the MCMC trajectory above after excluding the burn-in phase. 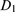 and 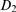 are in units of 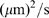. The gray vertical lines in (D.) mark the values of transition probabilities that were used for simulating the particle tracks. (E., F.) Typical errors and dispersions in maximum likelihood parameter estimates using the stochastic MCMC optimization scheme described in the text. Ten independent particle tracks consisting of 1000 steps each, sampled at 5 ms intervals were simulated with 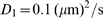, different values of 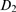, indicated by the colored dots in the left panel, 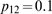 and 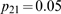. These parameter combinations correspond to the first four rows in Table S1. MCMC parameter estimates and 95% coverage intervals of parameter histograms are shown by the corresponding colored crosses that are centered at the maximum likelihood parameter values.

We then maximized this log likelihood with respect to the four model parameters to calculate their most likely values for a given set of tracks. We used a Markov Chain Monte Carlo (MCMC) algorithm (Algorithm 3; Fig. 4) to maximize the log likelihood function [26]. While it is computationally less efficient than traditional gradient-based maximization schemes, this algorithm is less liable to be stuck in a local maxima because of stochastic downhill steps. Moreover, by sampling the log likelihood landscape around the maxima, this algorithm establishes the measure of uncertainty in each parameter estimate. Fig. 5 (A and B) show a typical MCMC trajectory for fitting a set of simulated 2-state particle tracks to a 2-state HMM. There is an initial “burn-in” phase, indicated by the shaded region containing the first 20000 MCMC steps, during which the log likelihood increases nearly monotonically as the trajectory converges toward a maximum in log likelihood. After this burn-in phase, the log likelihood value and the parameter estimates maintain relatively steady values with small stochastic fluctuations. The distributions of parameter estimates from the MCMC optimization are shown in the histograms in Fig. 5C and D. We report the mean of each parameter distribution as the maximum likelihood parameter estimate and use the coefficient of variation (CV) to quantify the uncertainty in this estimate.

We assessed the MCMC parameter optimization scheme for a range of parameter values, using an ensemble of simulated tracks for each parameter set. The results, summarized in Table S1 (*Text S1*), include the maximum likelihood parameter estimates and their relative deviations from the true parameter values. For all but one parameter combination we tested, the maximum likelihood parameter estimates are remarkably close to their true values, with relative errors that are typically less than 10%. The error and dispersion in the parameter estimates are most appreciably affected by the relative magnitude of the two diffusion coefficients. In particular, as the two diffusion coefficients approach each other, the estimates of transition probabilities are progressively more error-prone and errors of as much as 70% arise. Notably, the magnitude of transition probabilities, either relative to each other - simulating a preferred state - or when they are uniformly high - simulating a frequent turnover of the particle between the two states - had only a minimal effect on the overall reliability of parameter estimates. We also tested the effects of varying the track length on the accuracy and variability of estimated parameters (Fig. S1, *Text S1*). As expected, both relative errors and dispersions in the parameter estimates decreased with an increasing number of frames.

In Fig. 5 (E and F), we plot another measure of dispersion in parameter estimates, namely, the span of a 95% coverage of the parameter distributions, which reveals any assymmetry in the parameter distributions. For fixed values of 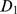, 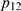 and 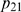, but varying 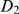 (corresponding to the first four parameter combinations in Table S1), we observe increasing error and dispersion as 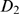 approaches 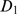. These trends arise because the log likelihood algorithm attempts to classify each displacement as arising either from 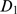 or 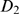, using equation 19. This classification is increasingly error-prone as the two states become indistinguishable, resulting in the errors seen in Table S1 and Fig. 5 (E and F). These results suggest that, if the maximum likelihood estimates of the two diffusion coefficients differ by less than two-fold, then the 2-state HMM is a poor descriptor of the system and parameter estimates (especially the transition probabilities) should be interpreted cautiously.

### Comparison of HMM and MSD analysis

**Figure 6:**
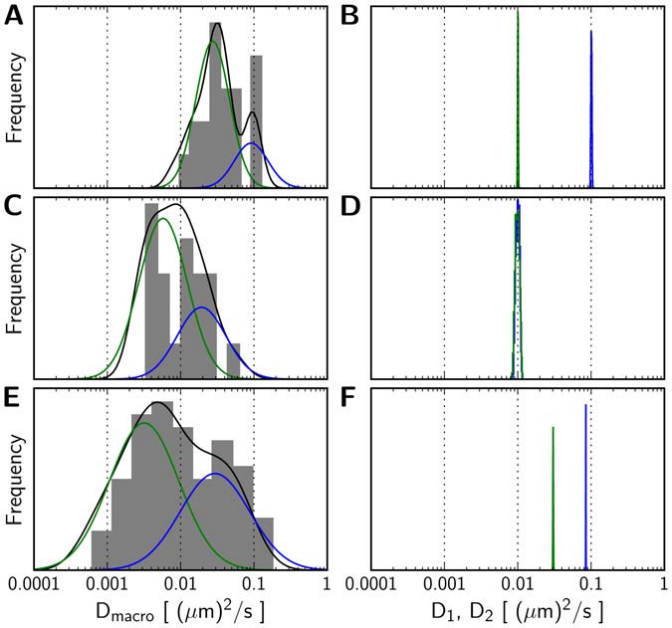
Comparison between MSD and HMM analysis. Distribution of 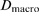 values estimated from MSD plots (left side, panels A,C,E) and the distribution of maximum likelihood parameter estimates for a 2-state HMM (right side, panels B,D,F), applied to simulated (top and middle, panels A,B and C,D) and experimental (bottom panels E,F) particle tracks. 20 simulated tracks each containing 1000 frames sampled at 100 frames/s were analyzed for the top and middle examples. The tracks used for the top example (panels A,B) were simulated for a 2-state system with parameters 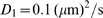, 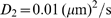, 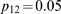 and 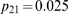, and the tracks used for the middle example (panels C,D) were simulated for pure Brownian diffusion with a diffusion coefficient of 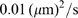. The tracks used for the bottom panels (E,F) are for TS-1/18-labeled LFA-1 in resting T cells, and consist of 75 individual tracks sampled for 4 s at 1000 frames/s [22]. For each track 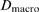 was calculated for 1/3 of the total length of the track. 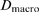 values for each set of tracks were binned and plotted as a histogram shown for each plot on the left. The corresponding densities of the distribution of 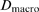 values were estimated and fitted to the sum of two lognormal distributions (shown in blue and green) as described previously [22].

The most commonly used analysis of single particle trajectories is to extract a diffusion coefficient from a linear fit to their mean squared displacement (MSD) over time [2]. Typically, a macroscopic diffusion coefficient, 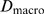, that captures the particle behaviour on a time scale of seconds is calculated. Heterogeneities in the distribution of 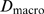 reveal multiple subpopulations of diffusing particles, and their relative contribution [22]. We used simulated particle trajectories to directly compare an MSD-based analysis with a 2-state HMM analysis over a range of frame rates, acquisition times and simulation parameters. Typical results are summarized in Fig. 6(A–D), with the output of 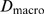 analysis shown on the left (Fig. 6A, C) and the output of a 2-state HMM analysis shown on the right (Fig. 6B, D). For simulated 2-state trajectories, we note that the distribution of 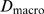 values is more dispersed than the individual distributions of 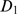 and 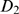 (Fig. 6A and B). Further, peaks of the two subpopulations constituting the 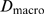 distribution do not accurately report the diffusion coefficients of the two underlying states. In contrast, the HMM analysis is less error-prone and yields sharper parameter distributions. Moreover, the distribution of 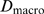 does not reveal the kinetics of the transition between the two states. Finally, we note that when trajectories are simulated with only a single underlying state, the 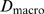 analysis shows spurious subpopulations with peaks flanking the true value of the single diffusion coefficient (Fig. 6 C), whereas the HMM analysis correctly reports a near complete overlap in the distributions of 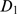 and 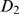, consistent with only a single identifiable diffusion coefficient (Fig. 6 D). These results offer additonal validation of the proposed HMM analysis for accurate resolution of 2-state dynamics that are not well-discerned with an MSD-based analysis of particle tracks.

### Analysis of LFA-1 particle trajectories

To test the applicability of the 2-state HMM described above, we analyzed a set of experimental SPT data for the T cell integrin, LFA-1. LFA-1 is critical for lymphocte adhesion and signaling, and has been previously studied using both SPT [22], [27]–[29] and FRAP techniques [30],[31]. In studies of LFA-1 lateral mobility on T cells, it has generally been observed that receptor diffusion is highly dependent upon cytoskeletal contacts. These interactions have manifested themselves in large immobile fractions and reduced diffusion coefficients. In previous work by Cairo et al., SPT experiments showed heterogeneous LFA-1 dynamics, with two apparent populations of diffusion coeffients [22]. The relative contributions to LFA-1 mobility from these two subpopulations were found to vary according to changes in the conformation of LFA-1 and the activation state of T cells. We sought to better understand the heterogeneity present in these experiments by analyzing them with the 2-state HMM model. A typical distribution of the most likely values of 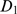 and 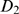 for one set of experiments is shown in Fig. 6 F, alongside the previously identified distribution of 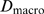 values segmented into the two subpopulations (Fig. 6 E). As was the case for simulated particle trajectories, the distribution of 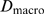 for LFA-1 is more dispersed with a significant overlap between the two subpopulations, compared to the distributions of 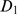 and 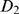 from the HMM analysis. However, it must be noted that unlike simulated trajectories, experimental particle tracks are subject to greater intrinsic variability arising from differences between individual cells. It is likely that this cell-to-cell variability is partly responsible for the observed dispersion in 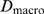 values, whereas the maximum likelihood parameter estimates from the HMM analysis essentially ignore this variability. Thus, for experimental particle tracks, the well-resolved peaks in the estimates of the diffusion coefficients (Fig. 6 F) should be interpreted as their most likely values over the population of cells analyzed, while an MSD-based analysis should be used to gauge the variability within the population.

**Figure 7:**
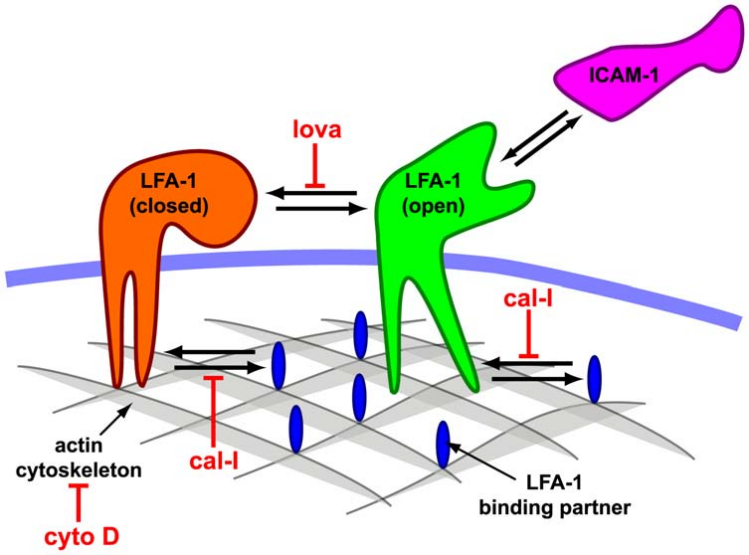
Schematic diagram of LFA-1 interactions and experimental conditions. A schematic diagram showing the putative interaction between LFA-1 and a binding partner (e.g. talin) associated with the actin cytoskeleton, and the pharmacological agents used to perturb the system. cyto D: cytochalasin D; lova: lovastatin; cal-I: calpain inhibitor I. Additionally, PMA was used to activate the cells. See reference [22] for details of treatment conditions.

We applied the 2-state HMM analysis to the data set of LFA-1 particle trajectories observed on T cells by Cairo et al. [22]. In these experiments, LFA-1 was labeled with either its cognate ligand ICAM-1, or an antibody, TS-1/18, known to block adhesion, and LFA-1 tracks were observed on resting cells, or those perturbed by various pharmacological agents (Fig. 7). Maximum likelihood parameter estimates for the 2-state model are reported in Table 1. In addition to these model parameters, we also list the stationary probabilities for the two states, a pseudo equilibrium constant 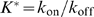 for the first order reaction (equation 2), and an effective diffusion coefficient, 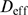 (equation 10), that captures the overall LFA-1 mobility for each set of particle tracks. The 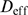 reported in Table 1 are nearly identical to 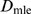 values calculated using equation 8, indicating that these two measures of the overall mobility of a particle are consistent with each other, and may be used interchangeably.

**Table 1:** 2-state HMM parameter estimates for LFA-1.

	Label	Treatment	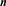	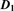		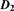		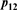	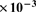	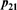	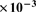				
				Mean	CV	Mean	CV	Mean	CV	Mean	CV	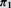	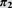	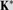	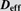
1.	TS1/18	untreated	75	0.085	0.38%	0.031	0.80%	4.2	6.9%	9.1	5.8%	0.68	0.32	0.46	0.068
2.	ICAM-1	untreated	38	0.081	0.43%	0.015	0.91%	3.9	7.7%	9.8	6.3%	0.72	0.28	0.40	0.062
3.	TS1/18	cyto D	36	0.082	0.42%	0.012	1.1%	3.4	8.2%	12	7.1%	0.78	0.22	0.27	0.067
4.	ICAM-1	cyto D	48	0.088	0.36%	0.019	1.2%	2.5	8.1%	11	6.9%	0.82	0.18	0.22	0.076
5.	TS1/18	PMA	39	0.082	0.38%	0.0038	0.78%	1.0	13%	4.2	11%	0.81	0.19	0.24	0.068
6.	ICAM-1	PMA	24	0.057	0.69%	0.0083	1.1%	19	5.2%	23	4.2%	0.55	0.45	0.81	0.035
7.	TS1/18	lova	42	0.086	0.39%	0.030	0.94%	1.1	15%	3.9	13%	0.78	0.22	0.28	0.074
8.	TS1/18	PMA+lova	42	0.090	0.40%	0.0053	0.54%	1.8	9.5%	3.4	8.0%	0.65	0.35	0.53	0.060
9.	TS1/18	cal-I	49	0.080	0.41%	0.013	1.9%	5.8	6.4%	29	4.8%	0.83	0.17	0.20	0.069
10.	TS1/18	PMA+cal-I	46	0.084	0.49%	0.012	0.51%	5.1	6.7%	4.3	5.9%	0.46	0.54	1.2	0.045

Maximum likelihood parameter estimates for a 2-state HMM applied to an ensemble of 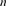 independent LFA-1 single particle tracks, labeled with beads coated with either ICAM-1 or the antibody TS-1/18, under various treatment conditions, as indicated (PMA: phorbol 12-myristate 13-acetate; cyto D: cytochalasin D; lova: lovastatin; cal-I: calpain inhibitor I). See reference [22] for details of treatment conditions and SPT setup. Derived parameters: 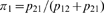 and 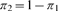 are stationary probabilities for the two states, and 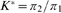 is a pseudo equilibrium constant. 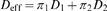 is an effective diffusion coefficient for the 2-state model. 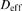 values reported here are nearly identical to the maximum likelihood diffusion coefficient 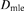 for a simple diffusion model, as defined in equation 8. All diffusion coefficients are reported in 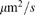.

We note that for all the experiments analyzed here, the maximum likelihood estimate of 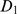 is at least double that of 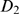, and typically greater by five-fold or more. This separation suggests relatively small errors in the parameter estimates (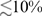), based on our tests of this analysis with simulated tracks of comparable length and sampling interval. Dispersions in the parameter distributions compare favourably with those for simulated tracks, with CV<2% for the two diffusion coefficients and CV<15% for the two transition probabilities. With the exception of ICAM-1-ligated LFA-1 in phorbol-12-myristate-13-acetate (PMA)-treated cells, the estimated value of 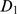 was 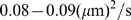, most likely capturing the diffusion of LFA-1 on the plasma membrane with relatively little interaction with the cytoskeleton. We observe a much greater variability in the estimates of 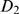, with values spanning nearly an order of magnitude, consistent with an active engagement between LFA-1 and the actin cytoskeleton in this state, thus rendering it susceptible to factors that affect this interaction, such as cytochalasin D treatment, or PMA-induced activation.

We observed that in untreated cells, ICAM-1 ligation reduces the overall mobility of LFA-1, compared to TS-1/18-labeled LFA-1, as assessed by the 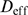 value for the two experiments (Table 1; cf. rows 1 and 2). This is consistent with the previously reported results using an MSD analysis [22], but the HMM analysis additionally reveals that the reduced mobility is primarily due to a two-fold decrease in 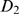, and not due to an increased fraction of time spent in the bound state. The decrease in 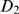 suggests that upon interaction with ICAM-1, the integrin may bind to an additional cytoskeletal-binding protein or could increase the number of cytoskeletal contacts as part of a cluster resulting in reduced mobility [32].

Treating cells with cytochalasin D reduces the lifetime of the bound state, with approximately 40% smaller 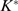 values compared to untreated cells (Table 1; cf. rows 1 and 3, and rows 2 and 4). Interestingly, this altered distribution between the two states is not reflected in a consistent trend in the overall mobility: 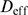 is virtually unchanged upon cytochalasin D treatment for the TS-1/18 label, but increases by nearly 20% for ICAM-1-treated cells. The difference arises because 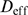 is affected by changes in both the two diffusion coefficients, as well as the relative lifetimes of the two states (equation 10). In this specific case, a marginal decrease in 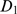 offsets the shift in the equilibrium to that state for TS-1/18-labeled LFA-1 such that the overall mobility is essentially unaltered upon cytochalasin D treatment. In contrast, for ICAM-1-ligated LFA-1, both diffusion coefficients increase upon cytochalasin D treatment (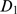 by nearly 10%, and 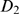 by over 25%), resulting in an increase in overall mobility. These results illustrate a significant advantage of the 2-state HMM analysis in its ability to capture subtle changes in multiple biophysical parameters, compared to an MSD-based analysis that only captures the overall mobility.

PMA-induced activation of T cells lowered 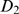 relative to its value in untreated cells, by over 8-fold for the TS-1/18 label (Table 1; cf. rows 1 and 5), and by nearly 2-fold for the ICAM-1 label (Table 1; cf. rows 2 and 6), albeit with important differences between the two cases. For cells labeled with TS-1/18, the reduced mobility of the bound state is offset by a shift in the equilibrium toward the free state, resulting in no net change in the overall mobility. In contrast, when LFA-1 is ligated with ICAM-1, and the cells are stimulated with PMA, the mobility of both free and bound LFA-1 are reduced and concurrently, there is a shift in the equlibrium toward the bound state, as seen by a two-fold increase in 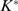. In combinations, these two factors dramatically lower the overall LFA-1 mobility resulting in the lowest 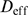 value across all the experiments analyzed here.

Notably, the combination of ICAM-1 ligation and PMA-induced activation also increases both the transition probabilities, 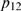 by nearly five-fold and 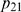 by over two-fold, relative to ICAM-1 ligation alone (Table 1; cf. rows 2 and 6). PMA-activation alone however reduced these transition probabilities relative to their values in resting cells labeled with TS-1/18, as well as decreasing 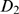 by nearly 10-fold. The transition probabilities are related to the on and off rates of the LFA-1 interaction with its cytoskeletal binding partners (equations 3 and 4). With the improved resolution of the HMM analysis, we can thus discern subtle regulatory mechanisms for the integrin receptors. It is clear that LFA-1 is tightly regulated by a dynamic interaction with its cytoskeletal binding partners. The effective diffusion of the receptor is likely controlled by altering the specific binding partner, or the on- or off-rates of the interaction. We see evidence for both these putative mechanisms: the decrease in 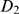 upon PMA-induced activation suggests that a different binding partner may be involved, whereas the increased transition probabilities upon the combination of ICAM-1 ligation and PMA treatment suggest that the turnover rate between the two states is altered. Thus, activation of the cell can alter either of the resolved diffusion coefficients or modify the equilibrium between the bound and free state. Together, these findings support the view that LFA-1 diffusion is a complex and dynamic process that integrates multiple biochemical cues, such as cellular activation, binding partner and conformational state, to influence T cell adhesion.

#### Conformation-dependent mobility of LFA-1

As previously noted using a 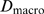 analysis, the diffusion profile of LFA-1 is conformation-dependent [22]. Lovastatin induces a conformational change in the I domain of LFA-1 that prevents the adoption of the active conformation required for ligand binding [33]. An MSD-based analysis showed that in cells treated with lovastatin, there was an increase in the mobile fraction relative to untreated cells. We could further resolve this result using our 2-state HMM analysis that shows that lovastatin does not alter 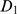 or 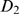, but instead shifts the equilibrium away from the bound state, thus increasing the overall receptor mobility (Table 1; row 7). Treating cells with PMA appeared to reverse this trend (Table 1; row 8).

Calpain is a cytosolic protease that cleaves the talin head domain, thus releasing LFA-1 from its cytoskeletal attachment site [34]. Thus, it is expected that inhibiting calpain would interfere with the exchange of LFA-1 between its free and bound states. Surprisingly, the 2-state HMM analysis of cells treated with the calpain inhibitor I (cal-I) shows a three-fold increase in 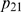, and a concurrent reduction the lifetime of the bound state (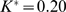, compared to 0.46 in untreated cells; cf. rows 1 and 9 in Table 1). Moreover, this trend was abrogated upon PMA treatment (Table 1; row 10), consistent with a previous observation that activation of LFA-1 by a calcium ionophore occurs independently from calpain-mediated cleavage [35].

### Segmentation of particle tracks

**Figure 8:**
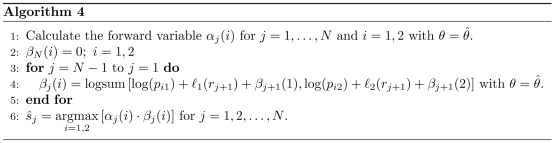
Forward-backward algorithm for identifying the most likely states of the particle for a given track 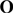.

The hidden Markov formulation that we used to analyze single particle tracks also allows us to identify the most likely state of the Markov chain at each step along a track. To achieve this, the forward-backward algorithm defines a backward variable
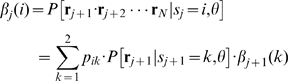
(14)that is the probability of observing the partial track 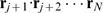 conditional on the particle being in state 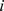 at the 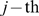 step and on model parameters 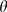. Therefore, the (unnormalized) probability of the particle being in state 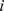 at step 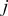, for an observed track 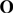 conditional on 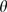 is
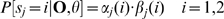
(15)where 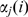 is defined in equation 12. The state 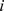 that maximizes this probability is the most likely state. We modified the recursive definition of 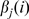 above for our likelihood-based calculation, as described in Algorithm 4 (Fig. 8), and estimated the most likely particle states for a given track, using the maximum likelihood parameter estimates, 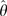, for the calculation.

**Figure 9:**
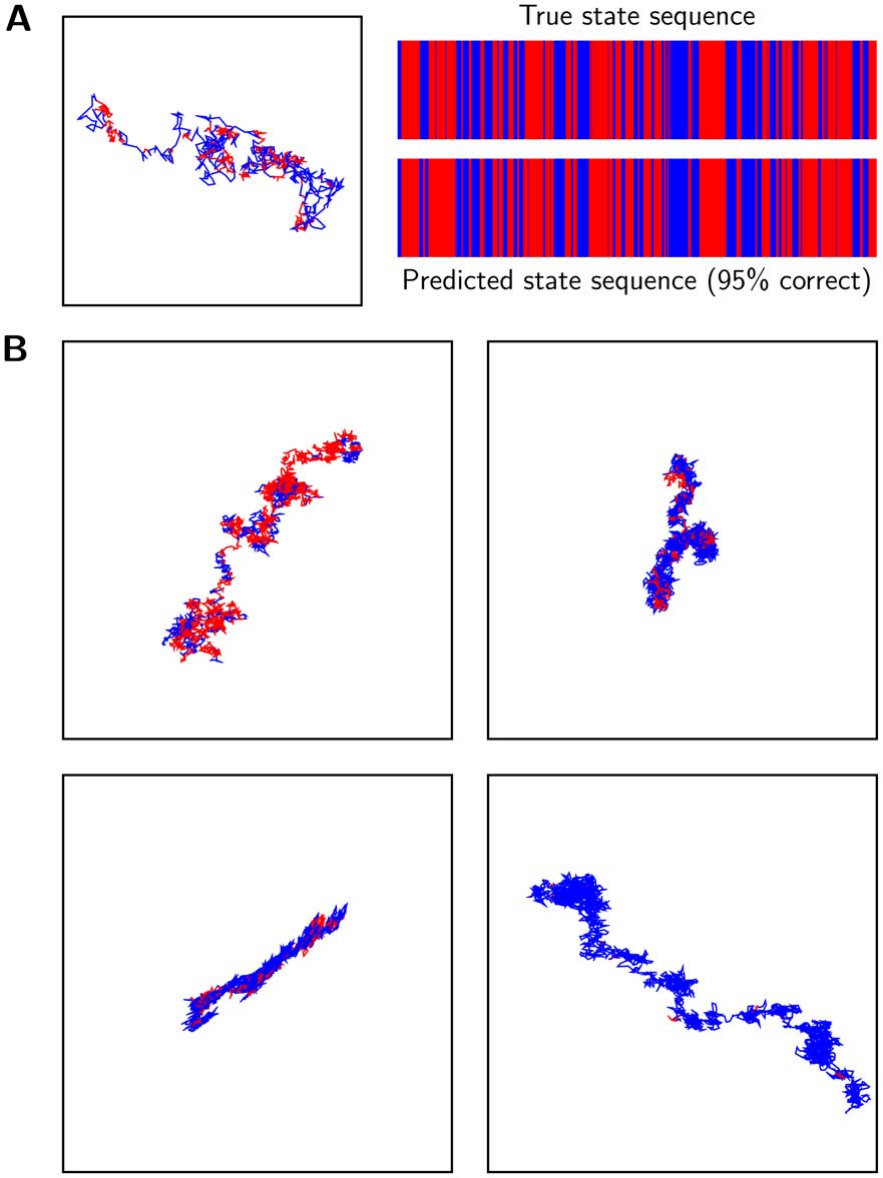
Segmentation of particle trajectories into the two hidden states. (A.) A simulated 2-state particle track with 1000 steps sampled at 5ms intervals, and parameters 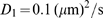, 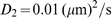 and 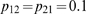, color coded to indicate the particle state (free: blue or bound: red). The state sequence is also depicted in the top bar code in the right panel, and the predicted state sequence, inferred using the track segmentation algorithm (Algorithm 4; Fig. 8), is shown in the bottom bar code. (B.) A selection of LFA-1 trajectories segmented into their two component states. Each enclosing box is a square of side 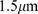.

We tested the performance of the segmentation algorithm for simulated trajectories that were previously used to assess the performance of the likelihood maximization algorithm (Table S1). For each set of trajectories, we used the maximum likelihood parameter estimates to identify the sequence of most likely particle state at each point along each track, and compared the prediction with the true identity of that state. Not surprisingly, the accuracy of track segmentation was strongly dependent on the accuracy of the maximum likelihood parameter estimates, and in turn on the separation between the two diffusion coefficients. When the diffusion coefficients differed by two-fold or greater, we could typically identify the true particle state more than 80% of the time. A representative simulated track, color-coded to identify the particle state at each point, is shown in Fig. 9 A, alongside the true and predicted state sequences for the trajectory depicted with state-sequence “barcodes” for an easy visual assessment of the segmentation.

**Figure 10:**
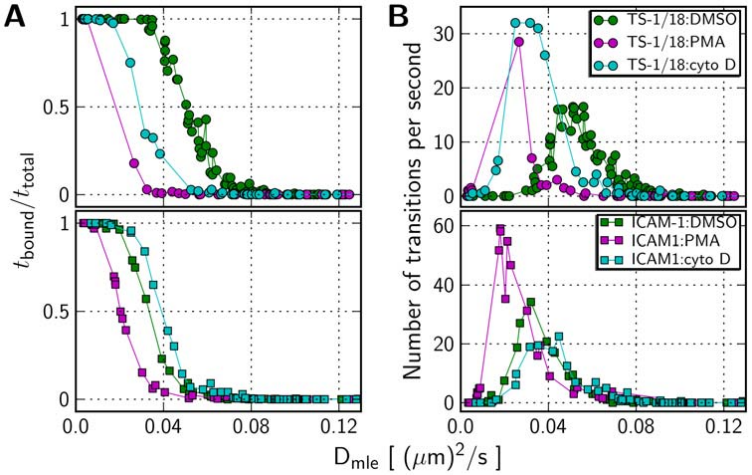
Relative fractions of time spent in each state. Classification of LFA-1 trajectories based on (A.) the fraction of total steps when the particle is in the bound state, and (B.) the mean number of transitions per second between the two states, plotted as a function of the overall mobility. The state sequence for each individual trajectory was established using the track segmentation algorithm with the maximum likelihood parameter estimates listed in Table 1. The overall mobility is indicated by 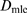 values calculated using equation 8 applied to each trajectory.

We applied the trajectory segmentation algorithm to LFA-1 particle tracks analyzed with a 2-state HMM. A selection of segmented LFA-1 particle tracks is shown in Fig. 9 B. We noted that for a majority of the observation period (4 s) the particles were found in a single state, suggesting relatively slow switching kinetics on the time scale of these experiments. To further classify the behavior of individual trajectories, we calculated the total number of state transitions during the 4 s data acquisition period, and the fraction of that time during which a particle was in the bound state(Fig. 10). The overall mobility of an individual particle decreased with increasing fractions of time in the second state, consistent with the smaller diffusion coefficient of the second state. Interestingly, these plots reveal that on the time scale of these experiments a majority of the particles were predominantly in a single state, and only a small number of trajectories had frequent state switches (Fig. 10B). This result is consistent with the generally small transition probabilities, typically 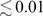, for this system (Table 1). It could also explain the relatively greater dispersion in the transition probability estimates reported here, as a substantial number of state switches would be required to estimate the transition probabilities accurately.

### Identification of the most optimal model

We now address the question of how to determine whether a 2-state model is indeed the best descriptor for the observed data, given one or more alternate models. We compared different models by means of Akaike's information criterion (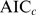, equation 21) and the associated Akaike weights (equation 23; see *Text S1* for details). We fitted simulated trajectories for pure Brownian diffusion with a 2-state model, and noted that for the maximum likelihood parameter estimates obtained in that case, the 2-state model effectively collapses to a single-state diffusion model (Fig. S2), that is preferred by the Akaike criterion. In contrast, when the trajectories are simulated from a 2-state process, the 2-state HMM outperforms a simpler 1-state model. Notably, for all LFA-1 trajectories analyzed here, the 2-state model is overwhelmingly preferred based on the Akaike criterion (data not shown), thus indicating the suitability of this model over a single state model to capture LFA-1 dynamics.

To determine whether a 2-state model is sufficient to describe the data, we attempted to further resolve the two states into component “sub-states”. After the intial segmentation of an ensemble of trajectories, we assembled all the displacements ascribed to 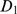 into a single trajectory, and likewise, all the diplacements ascribed to 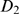 into another trajectory. The two resulting trajectories were then further analyzed with both a 1-state and a 2-state model. We found that it is indeed possible to further resolve the each of these trajectories with a 2-state model (Fig. S3), suggesting some heterogeneity in the two states originally identified. Importantly however, the separation between the diffusion coefficients of the sub-states is much smaller (approximately a factor of two) relative to the separation between the diffusion coefficients of the two original states (greater than an order of magnitude). As noted above, a small separation between the two diffusion coefficients implies that a 2-state model is an unreliable descriptor of the data. Thus, we conclude that our initial resolution of the data into two component states is sufficent to characterize the experimental trajectories. When this procedure was applied to an ensemble of simulated trajectories generated using the maximum likelihood parameter estimates for the data, we found that for the two virtual trajectories, the 2-state model effectively collapsed to a 1-state model.

## Discussion

In this study, we examined single particle trajectories for a membrane-associated protein that interacts with cytoskeletal binding proteins. Adhesion proteins at the cell membrane regulate a variety of biological phenomena including inflammation and antigen-presentation. Using a hidden Markov formulation to model 2D trajectories of a membrane protein, we outlined a systematic and easily-implemented procedure to parameterize a two-state model of diffusion and binding. Parameter estimates for this model can be used to identify the most probable state at each frame of the trajectory and thus divide it into mobile and immobile fragments. To establish the applicability of this analysis, we rigorously tested it with simulated trajectories for a range of parameter values. The HMM analysis revealed the diffusion coefficients of the individual states and identified transient state changes within single trajectories. Hidden Markov models have been previously used to analyze actomyosin and kinesin-microtubule movement data [36],[37], and DNA looping kinetics [38] in single-molecule microscopy experiments, but not to our knowledge, to analyze the lateral diffusion of membrane proteins. Thus, we have developed a novel methodology to analyze and interpret single particle trajectories of cell-surface molecules.

Our method expands upon the standard MSD analysis for SPT experiments, and provides previously inaccessible information about hetereogeneous diffusion. We are able to confidently detect the presence of two diffusion coefficients (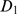 and 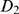), the transition probabilities for switching between these states (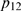 and 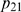), and an apparent equilibrium constant based on these probabilities (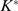). In previous studies of LFA-1 diffusion, a population-based MSD analysis was used to infer the presence of multiple states of diffusion. Our new analysis reveals that there are indeed two states responsible for the lateral-mobility of LFA-1, and that individual trajectories show a mixture of both states (Fig. 9). We are able to resolve the detailed state-switching behaviour of individual trajectories (Fig. 10). These values are accessible only in the aggregate using an MSD analysis, therefore, the method described here provides a new window into single-molecule experimental data. As noted above, the parameters provided by the HMM are inaccessible to a standard MSD analysis, and may be used to resolve changes in the identity or rate of specific interactions through changes in diffusion coefficients and transition probabilities, respectively.

We made two key simplifying assumptions: first, that the particle transitions between the two states with first order kinetics, and second, that all transitions occur at the sampling time. First order kinetics are justifiable when there is an excess of binding sites, but without direct experimental data, it is difficult to judge the merit of this assumption. Thus, the transition probabilities reported here must be interpreted with care, as they depend on 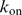, and therefore on the equilibrium substrate concentration, 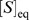. This caveat is especially important if transition probabilities reported here are used to derive first order on and off rates by solving equations 3 and 5 for 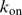 and 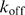. Nonetheless, given a measurement of the substrate concentration, and assuming that it doesn't change dramatically over the course of the 4 second particle track, our method could be used to estimate the true bimolecular on-rate for the interaction.

The assumption that transitions in the particle state occur on order of the sampling time is more easily justified in light of the relatively low transition probabilities that we observe (less than once every 100 frames). For infrequent transitions relative to the frame rate, the exact transition moment should not significantly alter our analysis. The validity of this assumption must be checked a-posteriori for a given experimental setup, by confirming that the transition probabilities are indeed small (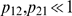) for the chosen frame rate. We plan to expand our analysis to the more general case when the transition rates are comparable to the acquisition frame rates and the transitions occur at intermediate times.

Our analysis offers some distinct advantages over an MSD-based approach. Firstly, by examining the diffusive behaviour of a particle at each step along a trajectory, heterogeneous diffusion is efficiently resolved. Secondly, unlike the distribution of 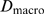 from an MSD analysis, the distributions of HMM parameter estimates quantify not only the diffusion coefficients of the underlying states, but also the kinetics of transitions between them. With some notable exceptions [39]–[41], these kinetic parameters are typically inaccessible in traditional analyses of SPT (or FRAP) experiments. There is mounting evidence that interprotein interactions affect the mobility of membrane proteins [42],[43], and some progress has been made toward modelling these effects [44]. In our analysis, we explicitly considered the effect of a binding interaction on the local diffusive behaviour of a molecule at short time scales and inferred the most likely parameter estimates for this interaction.

Of the two states identified in our analysis, the one with greater mobility (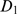) is most likely the freely diffusing form of LFA-1, with minimal interactions with intracellular proteins. This interpretation is well-supported by the relatively consistent value of 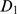 observed across a variety of experimental conditions (Table 1). The state with low mobility (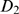), reported here and in a previous study [22], is likely to be either an actin cytoskeleton-associated form of LFA-1, or part of an integrin-associated signaling cluster that is slowly diffusing. Association with the actin cytoskeleton is strongly supported by the nearly twofold reduction in the pseudo-equilibrium constant, 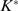, upon cytochalasin D treatment (Table 1). As well, a majority of the trajectories in cytochalasin D treated cells, are found predominantly in the high mobility state and exhibit very few state transitions (Fig. 10), suggesting that continued actin polymerization is required for maintaining the cytoskeletal attachment. Though the specific molecular mechanisms responsible are not fully understood, there is considerable evidence for a tightly regulated interaction between integrin receptors and the actin cytoskeleton, mediated by cytoskeletal proteins such as talin [45],[46]. Our technique thus offers the potential to resolve and quantify these interactions using SPT data for LFA-1.

We note that, the values of diffusion coefficients reported here are influenced by the use of a micron-sized bead to label the protein. The potential effects of a bead on the mobility of a membrane protein are discussed in reference [2], and include, enhanced drag due to the interaction between the bead and the extracellular matrix, and possible artifacts from crosslinking of the protein by the antibodies used. Nonetheless, the use of a bead allows for imaging at the high frame rates used in these experiments (1000 frames/s), thus exposing the transient state switching behavior that occurs on these short time scales.

Our analysis also assumes that the binding partner is homogeneously distributed, such that the transition probabilities have no spatial dependence. In this respect, it differs notably from another class of SPT analysis that has been used to resolve transient spatial confinement of particles [19]–[21]. Spatial confinement typically arises from the preferential partitioning of cell-surface receptors into or out of membrane microdomains. Such trapping or exclusion has been directly visualized for T cell signaling molecules with respect to CD2-enriched domains [43] and CD9 with respect to tetraspanin-enriched areas (TEA's) [41]. In another study, analysis of SPT data for a G-protein-coupled receptor showed evidence for confinement within domains that were themselves slowly diffusing (termed as “walking confined diffusion”) [42]. Our analysis does not directly resolve spatial confinement, but instead resolves heterogeneity in the temporal behavior of a diffusing particle. For sufficiently small confinement regions that are relatively uniformly distributed, the slow diffusing state in our model may indeed reflect the passage of a particle through such a confinement zone. But it is difficult to make such a conclusion in the absence of a secondary label used to visualize the membrane heterogeneity.

We have tested simulated 2-state trajectories and experimental LFA-1 trajectories using the spatial confinement algorithms described previously [19],[20], but do not find any consistent patterns between the temporal state-switching in our analysis and spatial confinement as identified by these algorithms (data not shown). This is not surprising, because these algorithms requires a clear separation between the macroscopic and microscopic diffusion coefficients for effective detection of confinement, and such separation is rarely observed in the LFA-1 data [22]. The LFA-1 trajectories were acquired with a very high frame rate (1000 frames/s), but for a relatively short interval (4 s). Consequently, these data are best suited for analyzing the behaviour of LFA-1 on a short time scale. This is in contrast with the typical acquisition rates of 30 frames/s or slower and acquisition times of tens of seconds that were used for the other studies cited above. These longer acquisition times allow the molecules to sample putative confinement regions and are therefore better suited to effectively distinguish short term diffusive behavior from long term confinement.

In general, analyzing spatial heterogeneity in mobility with the HMM formulation would require substantially more complex models than the one presented here, as the transition probabilities themselves would vary with the location of the particle. Additional complexity would be introduced by variations in the size of confinement regions. In future studies, we intend to examine modifications to our model that rigorously address these issues. A notable advantage of the present analysis is the lack of any user-tuned parameters, such as a characteristic confinement length (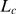) or a minimum segment length (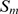), used in previous studies [19]. These parameters may vary for different experimental systems and their judicious choice is essential for succesfully detecting spatial confinement. In contrast, our analysis is directly applicable to a variety of experiments without requiring significant modification from its current form. However, we note that it may be possible to extract equivalents of the confinement length or other parameters from the results of the HMM analysis.

Finally, the likelihood-based approach that we adopted here is flexible and can be extended to account for other modes of motion. We tested a two-state Brownian model in this work, but the HMM approach could be used to introduce additional states or alternative models of mobility, such as directed motion. This approach has the potential to resolve extremely complex and heterogeneous trajectories. The use of likelihood as a metric for the quality of a model allows for statistically well-defined comparisons between various models, using 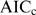, as described here, and other tests described elsewhere [47]. In summary, we believe that fitting particle tracking data to a well-defined model and using likelihood maximization to estimate model parameters is a natural and powerful tool for inferring and quantifying the spatiotemporal dynamics of cell surface proteins.

## Materials and Methods

### LFA-1 labeling and single-particle trajectories

Experimental LFA-1 trajectories used were acquired as described in Cairo et al [22]. Briefly, 1 micron beads were labeled with either an adhesion protein (ICAM-1) or a Fab fragment of an LFA-1 binding antibody (TS1/18). The beads were then blocked to prevent non-specific binding, and Jurkat T cells (clone E6.1, ATCC, Manassas, VA, USA) were labeled with beads and observed using video microscopy [48]. Cells were treated with HBSS buffer containing either a vehicle control (DMSO), phorbol-12-myristate-13-acetate (PMA), cytochalasin D (cytoD), or calpain inhibitor-I (cal-I). Trajectories were collected on live cells at 1000 FPS (1 ms) and converted to trajectories using Metamorph (Universal Imaging, Downington, PA, USA). Data were analyzed by either an MSD algorithm combined with a population analysis [22] or by the HMM method described here.

### Simulation of single particle tracks

For a particle undergoing Brownian diffusion in a 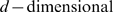 space with a diffusion coefficient 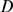, the probability density of observing a displacement 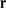 after a time interval 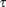 is given by:
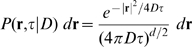
(16)


In this study we are concerned with single particle tracks of a membrane-associated protein that is imaged at fixed time intervals. Thus, 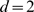 and 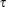 is the frame rate at which the particle is imaged. A simulated track therefore consists of 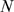 successive displacements, 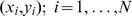 with the displacement along each dimension distributed normally, with mean 0 and variance 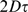. To simulate Brownian diffusion, we used the Matlab function normrnd to generate such a sequence of displacements and then cumulatively summed them to calculate the particle coordinates.

To simulate trajectories for a particle with 2-state diffusion we first generated a Markov chain 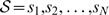 where 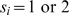 denotes the state of the particle at the 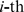 time point. The 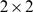 Markov transition matrix
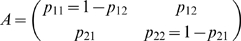
(17)is composed of the probabilities 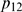 and 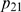 for transitions between the two states. The Markov chain was simulated using Algorithm 1 (Fig. 1). The particle displacements 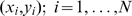 were then drawn randomly from a normal distribution with 0 mean and a variance 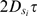.

### Hidden Markov model likelihood estimation

A particle trajectory consists of a sequence of individual displacements, denoted as 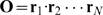, where, 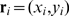. We calculated the log likelihood, 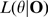, of parameter values 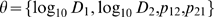 for a particle track as described next. First, we defined the likelihood of a diffusion coefficient 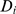 for an individual displacement 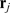 as
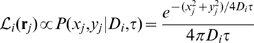
(18)and the corresponding log likelihood as
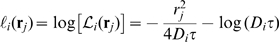
(19)where 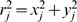. The proportionality in the first equation arises because the likelihood function is only defined up to an arbitrary multiplicative constant. Likewise, the log likelihood function is only defined to an arbitrary additive constant, and in our definition (equation 19) we only retained terms that contain an explicit dependence on model parameters, ignoring coefficient such as 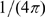. The log likelihood of the parameters for a sequence of displacements was calculated using Algorithm 2 (Fig. 3), which is a modified version of the forward-backward algorithm [24],[49]. Finally, the log likelihood function for an ensemble of independent trajectories, 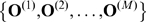, is simply the sum of the log likelihood function evaluated for each trajectory.
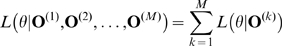
(20)


### Maximum likelihood parameter estimation

To estimate the maximum likelihood parameters of a 2-state HMM for a set of tracks, we used a stochastic Markov Chain Monte Carlo (MCMC) optimization scheme (Algorithm 3; Fig. 4). This algorithm assigns random initial values to all the parameters and iteratively traverses the parameter space through a succession of small displacements along each parameter axis. For each proposed displacement, the log likelihood function is evaluated at parameter values after the displacement and compared to the log likelihood for the current parameter values. A proposed displacement is accepted or rejected using a Metropolis rejection scheme: any proposed displacement that increases the log likelihood from its current value is accepted, but a proposed displacement that decreases the log likelihood from its current value is only accepted with a probability equal to the fractional change in the likelihood function after the proposed move. Typically the MCMC runs were 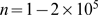 steps long with an initial burn-in phase during which the MCMC trajectories approach an equilibrium. The scales of displacement, 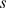, were adjusted to achieve an acceptance rate of 20–40% along each parameter axis after the burn-in phase. The acceptance rate is defined to be the ratio of number of accepted moves to the total number of proposed moves along a parameter axis during the MCMC run. The sample means of the MCMC trajectories, after excluding the burn-in phase, were reported as the maximum likelihood parameter estimates. We also calculated the coefficient of variation (CV), the ratio of the sample standard deviation to the sample mean, to measure the variability of the parameter estimates.

### Track segmentation

We define 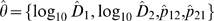 as the set of maximum likelihood parameters for a given track 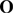 and use a modified version of the forward-backward algorithm to estimate the most likely state, 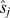 of the Markov chain at each step along the track (Algorithm 4; Fig. 8).

### Model comparison

To compare the effectiveness of different models in describing a set of tracks, we used the Akaike information criterion (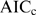), defined as
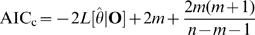
(21)where 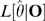 is the log likelihood function of the maximum likelihood parameter set 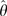 for a model with 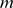 parameters, given 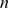 independent observations. Here, 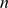 is the number of individual displacements in the trajectory 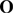. To interpret the 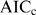 values for different models, we use the rescaled 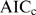 values, defined as
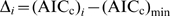
(22)where 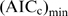 is the minimum 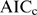 value among all models under consideration, that is, 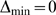. Each model is then assigned an Akaike weight
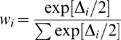
(23)that measures the relative evidence in its favour. The sum in the denominator is over all the models under consideration [47].

## Supporting Information

Text S1 Supporting information text.
(0.12 MB PDF)

Table S1 Maximum likelihood parameter estimates for simulated 2-state trajectories.
(0.03 MB PDF)

Figure S1 Accuracy of parameter estimates as a function of trajectory length.
(0.05 MB PDF)

Figure S2 Pure Brownian diffusion analyzed with a two-state HMM.
(0.62 MB PDF)

Figure S3 Analysis of segmented trajectories.
(0.04 MB PDF)
