# Supplementary material for: A Hidden Markov Model for Single Particle Tracks Quantifies Dynamic Interactions between LFA-1 and the Actin Cytoskeleton
Source: PLoS Comput Biol. 2009 Nov 6;5(11):e1000556. doi: 10.1371/journal.pcbi.1000556 (PMC2768823; doi:10.1371/journal.pcbi.1000556)
Supplement: Text S1 — Supporting information text. (0.12 MB PDF) [file pcbi.1000556.s001.pdf]

Supplementary Text S1

for

"A Hidden Markov Model for Single Particle Tracks Quantifies Dynamic Interactions between LFA-1 and the Actin Cytoskeleton"

Raibatak Das, Christopher W. Cairo and Daniel Coombs.

Derivation of transition probabilities for the 2-state model

Here, we derive the relationship between the first order reaction rate constants, k_{on} and k_{off}, and the Markovian transition probabilities, p_{12} and p_{21} (equations 3-5 in main text). We define $p_{12}(t)$ to be the probability that a particle that is initially in state 1 (i.e. at time 0) is found in state 2 at a later time $t > 0$. The probabilities $p_{11}(t)$, $p_{21}(t)$ and $p_{22}(t)$ are defined analogously. Also, by definition,

$$p_{11}(t) = 1 - p_{12}(t) \quad \text{and} \quad p_{22}(t) = 1 - p_{21}(t). \tag{S1}$$

The first order transitions $D_1 \xrightarrow{k_{\text{on}}} D_2$ and $D_2 \xrightarrow{k_{\text{off}}} D_1$ are exponential processes with characteristic times, $1/k_{\text{on}}$ and $1/k_{\text{off}}$, respectively. Therefore, for a small interval δt,

$$p_{12}(\delta t) \simeq k_{\text{on}}\ \delta t \quad \text{and} \tag{S2}$$

$$p_{21}(\delta t) \simeq k_{\text{off}}\ \delta t. \tag{S3}$$

Next, consider a time interval $t + \delta t$, for which

$$p_{12}(t+\delta t) = p_{11}(t) \cdot p_{12}(\delta t) + p_{12}(t) \cdot p_{22}(\delta t) \tag{S4}$$

$$\simeq [1 - p_{12}(t)] \cdot k_{\text{on}}\delta t + p_{12}(t) \cdot [1 - k_{\text{off}}\delta t]. \tag{S5}$$

It follows that:

$$\frac{dp_{12}}{dt} = \lim_{\delta t \to 0} \frac{p_{12}(t+\delta t) - p_{12}(t)}{\delta t} = -(k_{\text{on}} + k_{\text{off}})p_{12} + k_{\text{on}} \tag{S6}$$

with the initial condition $p_{12}(0) = 0$. This differential equation is easily solved using the integrating factor $e^{(k_{\text{on}}+k_{\text{off}})t}$, to obtain equation 3:

$$e^{(k_{\text{on}}+k_{\text{off}})t}\ \frac{dp_{12}}{dt} + (k_{\text{on}} + k_{\text{off}})\ e^{(k_{\text{on}}+k_{\text{off}})t}\ p_{12} = k_{\text{on}} e^{(k_{\text{on}}+k_{\text{off}})t}$$

$$\int_{t=0}^{t=\tau} d\left[e^{(k_{\text{on}}+k_{\text{off}})t} p_{12}\right] = k_{\text{on}} \int_0^\tau e^{(k_{\text{on}}+k_{\text{off}})t} dt$$

$$e^{(k_{\text{on}}+k_{\text{off}})\tau} p_{12}(\tau) = \frac{k_{\text{on}}}{k_{\text{on}} + k_{\text{off}}} \left[e^{(k_{\text{on}}+k_{\text{off}})\tau} - 1\right]$$

$$p_{12}(\tau) = \frac{k_{\text{on}}}{k_{\text{on}} + k_{\text{off}}} \left[1 - e^{-(k_{\text{on}}+k_{\text{off}})\tau}\right]. \tag{S7}$$

By symmetry, switching k_{on} and k_{off} in the above expression leads to the equation for $p_{21}(\tau)$ (equation 4).

Parameter estimates for simulated tracks

In Table S1 maximum likelihood parameter estimates from fitting simulated 2-state trajectories are shown for a range of simulation parameters. In Fig. S1 error and dispersion in parameter estimates are plotted as a function of the trajectory length.

Identification of optimal model

We simulated a set of Browninan diffusion trajectories and analyzed them with a 1-state as well as a 2-state model. As expected, D_{mle}, for a 1-state model recovered the diffusion coefficient. When analyzed with a 2-state model, we observed one of two behaviours shown in Figure S2. Either estimates for both D_1 and D_2 coverged to the same value while the transition probabilities did not converge to any particular value (Fig. S2A, B), or, less frequently, only one of the two diffusion coefficients converged to the true diffusion coefficient, and the transition probability leading out of that state converged to a very small number (Fig. S2C, D). In other words, the state with the convergent diffusion coefficient essentially behaved as an absorbing state. Either of these outcomes implied that, on the timescale of the track acquisition, the 2-state model essentially collapsed to a 1-state model. Based on the Akaike criterion, the 2-state model was rejected because of the greater number of parameters relative to a 1-state model. In contrast, the 2-state HMM, when applied to a set of 2-state trajectories, accurately recovered the simulation parameters, and was judged significantly better than a 1-state model, on the basis of their Akaike weights.

Next, we segmented an ensemble of LFA-1 trajectories using a 2-state HMM and separately analyzed the particle displacements belonging to each state using a 2-state model (Figure S3; see text for details).
