# Supplementary material for: A Hidden Markov Model for Single Particle Tracks Quantifies Dynamic Interactions between LFA-1 and the Actin Cytoskeleton
Source: PLoS Comput Biol. 2009 Nov 6;5(11):e1000556. doi: 10.1371/journal.pcbi.1000556 (PMC2768823; doi:10.1371/journal.pcbi.1000556)
Supplement: Table S1 — Maximum likelihood parameter estimates for simulated 2-state trajectories. (0.03 MB PDF) [file pcbi.1000556.s002.pdf]

| Simulation values | | | | Maximum likelihood estimates | | | | | | | | | | | | Correct state id. |
|---|---|---|---|---|---|---|---|---|---|---|---|---|---|---|---|---|
| | | | | $D_1$ | | | $D_2$ | | | $p_{12}$ | | | $p_{21}$ | | | |
| $D_1$ | $D_2$ | $p_{12}$ | $p_{21}$ | Mean | RE | CV | Mean | RE | CV | Mean | RE | CV | Mean | RE | CV | (%) |
| 0.1 | 0.01 | 0.1 | 0.05 | 0.101 | 1.4% | 2.4% | 0.0100 | 0.0% | 1.6% | 0.102 | 1.9% | 8.2% | 0.0491 | 1.8% | 5.6% | 96±1 |
| | 0.03 | | | 0.0994 | 0.6% | 3.0% | 0.0297 | 1.0% | 2.1% | 0.0953 | 4.7% | 14% | 0.0526 | 5.2% | 9.6% | 89±3 |
| | 0.05 | | | 0.102 | 1.8% | 4.3% | 0.0503 | 0.6% | 2.8% | 0.102 | 2.4% | 18% | 0.0455 | 9.0% | 18% | 81±3 |
| | 0.08 | | | 0.0914 | 8.6% | 3.0% | 0.0665 | 17% | 16% | 0.169 | 69% | 70% | 0.0384 | 23% | 36% | 35±6 |
| 0.1 | 0.01 | 0.1 | 0.1 | 0.0999 | 0.1% | 2.1% | 0.0103 | 3.0% | 2.1% | 0.101 | 1.4% | 7.0% | 0.0960 | 4.0% | 5.1% | 94±1 |
| | | | 0.3 | 0.101 | 1.2% | 1.8% | 0.0105 | 5.0% | 4.2% | 0.104 | 3.5% | 6.8% | 0.296 | 1.3% | 4.7% | 90±1 |
| | | | 0.5 | 0.0997 | 0.3% | 1.7% | 0.0096 | 4.0% | 7.3% | 0.110 | 9.9% | 7.8% | 0.543 | 8.6% | 2.2% | 88±2 |
| | | | 0.9 | 0.100 | 0.0% | 1.7% | 0.0086 | 14% | 16% | 0.0977 | 2.3% | 14% | 0.944 | 4.9% | 2.9% | 90±1 |
| 0.1 | 0.01 | 0.5 | 0.5 | 0.0982 | 1.8% | 2.2% | 0.0097 | 3.0% | 2.9% | 0.510 | 2.1% | 2.6% | 0.527 | 5.5% | 1.8% | 85±2 |
| | | 0.9 | 0.9 | 0.0988 | 1.2% | 1.9% | 0.0102 | 2.0% | 2.1% | 0.904 | 0.4% | 1.0% | 0.904 | 0.4% | 0.9% | 95±1 |

**Table S1.** Maximum likelihood parameter estimates for simulated tracks analyzed with a 2-state HMM, and the accuracy of maximum likelihood particle states as identified by the track segmentation algorithm. For each parameter combination listed, 10 independent tracks, 1000 steps each, with a sampling interval of 5 ms were simulated and maximum likelihood parameters for a 2-state HMM were calculated with an MCMC optimization scheme. RE = Relative error of the maximum likelihood estimate with respect to the parameter value used for the simulation. CV = Coefficient of variation, defined as the ratio of the standard deviation to the mean for the parameter distribution obtained from the MCMC trajectory. Most likely particle states for each track were determined using the track segmentation algorithm and compared with the actual state sequence of the corresponding Markov chain. The fraction of time when the particle state is correctly identified is reported as mean ± standard deviation for each set.
