# Supplementary material for: A Hidden Markov Model for Single Particle Tracks Quantifies Dynamic Interactions between LFA-1 and the Actin Cytoskeleton
Source: PLoS Comput Biol. 2009 Nov 6;5(11):e1000556. doi: 10.1371/journal.pcbi.1000556 (PMC2768823; doi:10.1371/journal.pcbi.1000556)
Supplement: Figure S1 — Accuracy of parameter estimates as a function of trajectory length. (0.05 MB PDF) [file pcbi.1000556.s003.pdf]

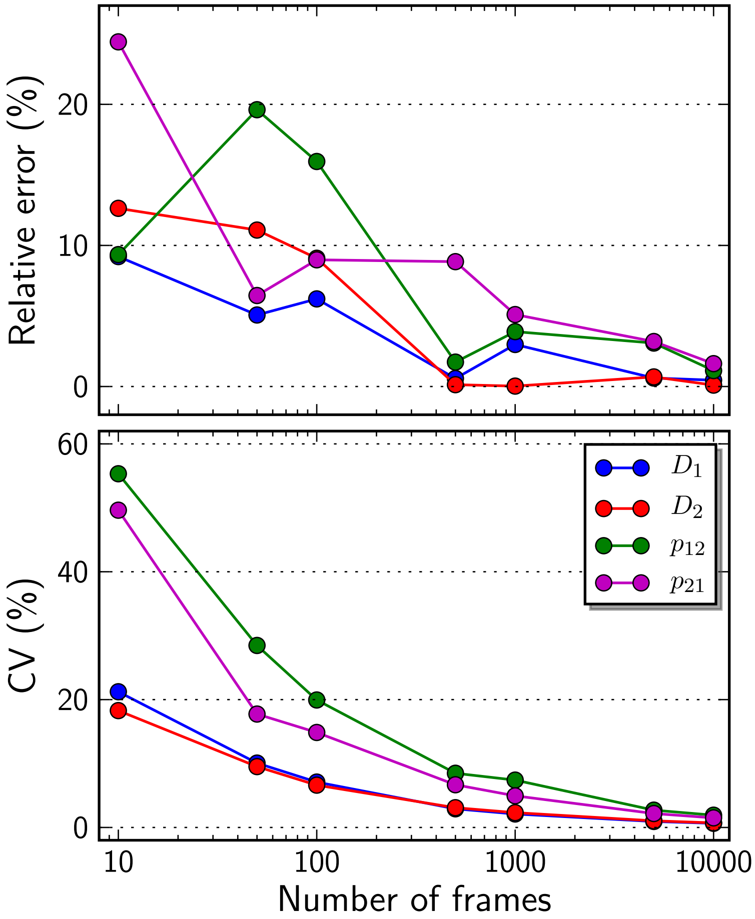

Figure S1. Accuracy of parameter estimates as a function of trajectory length. To examine the effect of trajectory length on the accuracy and variability of maximum likelihood parameter estimates, we simulated a set of 10 independent particle trajectories, each containing 10000 frames, sampled at 5 ms intervals with parameters $D_1 = 0.1\ (\mu\text{m})^2/\text{s}$, $D_2 = 0.01\ (\mu\text{m})^2/\text{s}$, and $p_{12} = p_{21} = 0.1$. The full length trajectories were truncated at specific lengths indicated on the x-axis, and the maximum likelihood parameter estimates were calculated from the truncated trajectories. The relative error (top panel) and dispersion (bottom panel) in the maximum likelihood parameter estimates are plotted as a function of the truncation length.
