# Supplementary material for: A Hidden Markov Model for Single Particle Tracks Quantifies Dynamic Interactions between LFA-1 and the Actin Cytoskeleton
Source: PLoS Comput Biol. 2009 Nov 6;5(11):e1000556. doi: 10.1371/journal.pcbi.1000556 (PMC2768823; doi:10.1371/journal.pcbi.1000556)
Supplement: Figure S2 — Pure Brownian diffusion analyzed with a two-state HMM. (0.62 MB PDF) [file pcbi.1000556.s004.pdf]

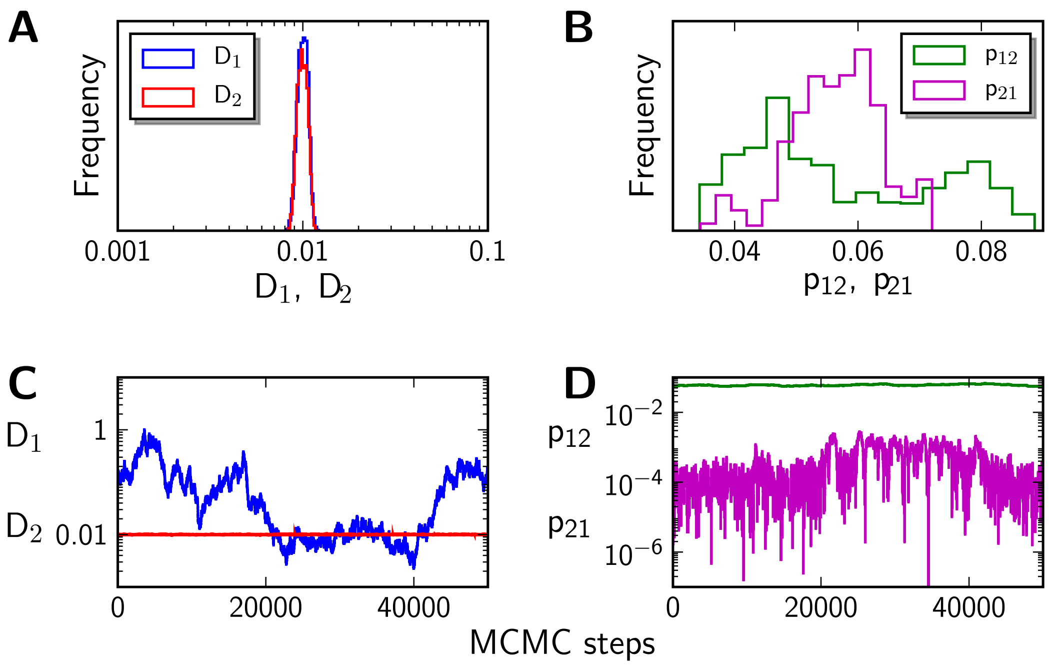

Figure S2. Pure Brownian diffusion analyzed with a two-state HMM. 10 independent trajectories with 1000 steps each were simulated with a diffusion coefficient of 0.01 $(\mu m)^2/s$, and fitted to a 2-state HMM. As discussed above, the 2-state HMM collapses to a 1-state model in one of two possible ways: Either the two diffusion coefficients converge to the same value (A. and B.), or one of the states is inaccessible because of a very small transition probability leading into it (C. and D.) D_1 and D_2 are in units of $(\mu m)^2/s$.
