# Supplementary material for: A Hidden Markov Model for Single Particle Tracks Quantifies Dynamic Interactions between LFA-1 and the Actin Cytoskeleton
Source: PLoS Comput Biol. 2009 Nov 6;5(11):e1000556. doi: 10.1371/journal.pcbi.1000556 (PMC2768823; doi:10.1371/journal.pcbi.1000556)
Supplement: Figure S3 — Analysis of segmented trajectories. (0.04 MB PDF) [file pcbi.1000556.s005.pdf]

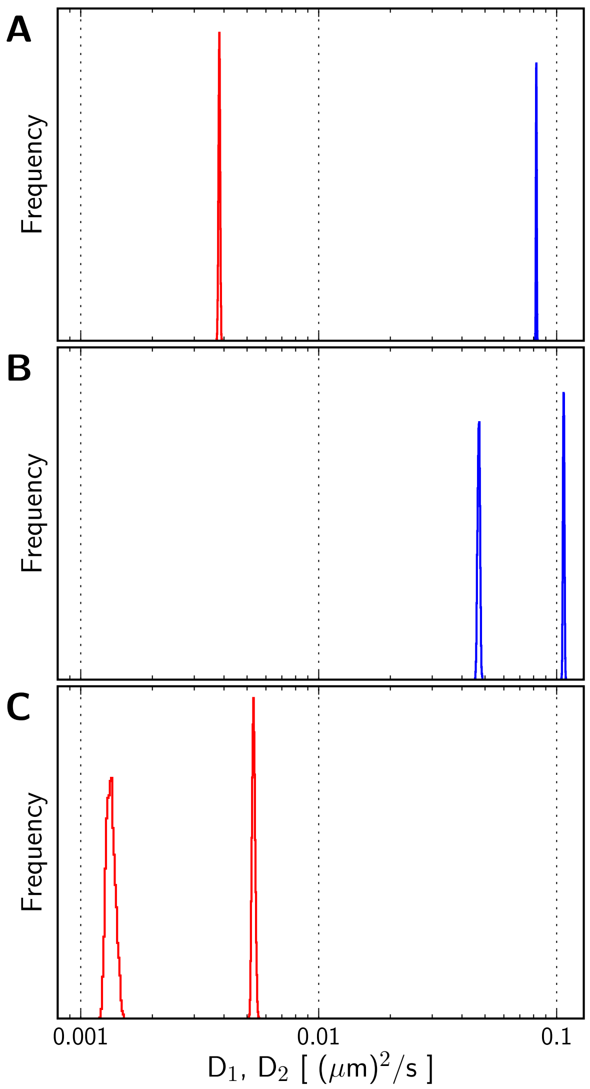

Figure S3. Analysis of segmented trajectories. (A.) An ensemble of experimental LFA-1 trajectories were analyzed with the 2-state HMM to identify parameter distributions for the two diffusion coefficients, D_1(blue) and D_2(red). The most probable state sequence for each trajectory was determined using Algorithm 4 and all segments belonging to each state were concatenated into a single trajectory. (B, C.) The two resulting trajectories, one containing all displacements attributed to D_1 (B), and the other containing all displacements attributed to D_2 (C) in the original analysis were then further analyzed with a 2-state HMM.
